# Phenotype clustering of breast epithelial cells in confocal images based on nuclear protein distribution analysis

**DOI:** 10.1186/1471-2121-8-S1-S3

**Published:** 2007-07-10

**Authors:** Fuhui Long, Hanchuan Peng, Damir Sudar, Sophie A Lelièvre, David W Knowles

**Affiliations:** 1 Life Sciences Division, Lawrence Berkeley National Laboratory, Berkeley, CA 94720 USA; 2 Genomics Division West, Lawrence Berkeley National Laboratory, Berkeley, CA 94720 USA; 3 Department of Basic Medical Science, Purdue University, West Lafayette, IN 47907 USA; 4 Janelia Farm Research Campus, Howard Hughes Medical Institute, Ashburn, VA 20147 USA

## Abstract

**Background:**

The distribution of chromatin-associated proteins plays a key role in directing nuclear function. Previously, we developed an image-based method to quantify the nuclear distributions of proteins and showed that these distributions depended on the phenotype of human mammary epithelial cells. Here we describe a method that creates a hierarchical tree of the given cell phenotypes and calculates the statistical significance between them, based on the clustering analysis of nuclear protein distributions.

**Results:**

Nuclear distributions of nuclear mitotic apparatus protein were previously obtained for non-neoplastic S1 and malignant T4-2 human mammary epithelial cells cultured for up to 12 days. Cell phenotype was defined as S1 or T4-2 and the number of days in cultured. A probabilistic ensemble approach was used to define a set of consensus clusters from the results of multiple traditional cluster analysis techniques applied to the nuclear distribution data. Cluster histograms were constructed to show how cells in any one phenotype were distributed across the consensus clusters. Grouping various phenotypes allowed us to build phenotype trees and calculate the statistical difference between each group. The results showed that non-neoplastic S1 cells could be distinguished from malignant T4-2 cells with 94.19% accuracy; that proliferating S1 cells could be distinguished from differentiated S1 cells with 92.86% accuracy; and showed no significant difference between the various phenotypes of T4-2 cells corresponding to increasing tumor sizes.

**Conclusion:**

This work presents a cluster analysis method that can identify significant cell phenotypes, based on the nuclear distribution of specific proteins, with high accuracy.

## Background

Histological classification of biopsied breast tissue plays a key role in mammary cancer detection and in determining patient treatment. Current methods rely on gross signatures of cellular and tissue organization including tubular formation, nuclear pleomorphism and mitotic activity. To aid the early detection and diagnosis of mammary tumors, quantitative techniques are highly needed that could not only help automate the classification process but also provide subcellular information that could be used to reveal new subclasses of tumor within each pathological grade.

Increasing evidence has shown that chromatin-associated proteins are important in directing nuclear functions involved in the control of cell proliferation and differentiation [1-3]. Using tissue models, formed by culturing human mammary epithelial cells (HMECs) from the HMT-3522 cancer progression series in Matrigel™ (3D culture), earlier studies showed that the distribution of Nuclear Mitotic Apparatus (NuMA) protein was remarkably different in non-neoplastic cells that were proliferating compared to those that had completed acinar morphogenesis by forming polarized glandular tissue structures [4]. For instance, during the 10-day in vitro morphogenesis process, NuMA staining was reported as diffusely distributed within the nuclei of proliferating cells, and had aggregated into foci of increasing size as cells arrested proliferation and completed acinar morphogenesis [4].

Based on these findings, Knowles et al then developed an image-based technique, called local bright feature (LBF) analysis [5]. The technique uses fluorescence images of total DNA and specifically stained nuclear proteins and calculates the radial distribution of the density of bright immunostained features as a function of the distance from the perimeter of the nucleus to its center. The LBF analysis was used to quantify the distribution of fluorescently stained NuMA from confocal images of non-neoplastic (S1) and malignant (T4-2) HMT-3522 HMECs, cultured in 3D for up to 12 days [5]. By averaging the LBF distributions over populations of cells with the same phenotype, the study showed that the LBF analysis reproducibly captured changes in NuMA distribution along the morphogenic process in non-neoplastic S1 cells. It also revealed that the NuMA distribution in malignant T4-2 cells was diffuse and independent of the number of days the cells were in culture [5].

Here we report a cluster analysis approach, based on the distribution of nuclear proteins, that robustly calculates the statistical significance between cell phenotypes, which are defined by the behavior of the cells in 3D culture. The method first groups LBF distributions into clusters using multiple traditional clustering methods. The results are then combined by a probabilistic ensemble approach into a set of consensus clusters that can be used to reliably define all possible LBF distributions that exist within a data set. This then allows cluster histograms to be computed which show how the LBF distributions in individual cells from a group are distributed over the consensus clusters. These cluster histograms represent a new way of linking the phenotype of groups of phenotypically similar cells, defined by their behavior in 3D culture, with their LBF distributions, quantified microscopically. Further, by grouping the LBF cluster histograms in multiple ways, the method is then able to build a phenotype tree and to calculate the statistical significance between each grouping. Each level of the tree corresponds to a different phenotype division of the cells and provides a way to predict which of the cell phenotypes, or grouping of cell phenotypes are significantly different from each other. These methods were then applied to the LBF distributions of NuMA in S1 and T4-2 cells, previously reported in Knowles et al [5]. The resulting cluster histograms clearly showed that the distribution of NuMA changes during the morphogenic process as non-neoplastic S1 cells growth arrest and differentiate. The resulting phenotype tree showed that non-neoplastic S1 cells could be distinguished from malignant T4-2 cells with 94.19% accuracy; that proliferating S1 cells could be distinguished from differentiated S1 cells with 92.86% accuracy; and clearly indicated that NuMA distribution was unchanged in the various phenotypes of malignant T4-2 cells.

## Results

### Dataset

As described in [5], non-neoplastic HMT-3522 S1 cells were cultured in 3D in the presence of Matrigel™ for up to 12 days to induce acinar morphogenesis. Malignant HMT-3522 T4-2 cells were cultured under similar conditions for a maximum of 11 days to avoid the overgrowth of tumor nodules. DNA was stained with DAPI to visualize the limits of the nuclear volume and NuMA proteins were labeled with Texas red. Three-dimensional images were acquired using a Zeiss 410 confocal laser-scanning microscope with planapochromatic 63×, 1.4 numerical aperture lens. The resulting voxel dimensions of the 3D images were 0.08 × 0.08 μm in the plane of the slide and 0.5 μm along the optical direction.

We used three image datasets to test our phenotype clustering approach. The first dataset contains 2673 non-neoplastic S1 cells taken from 77 confocal images. Images 1–25, 26–45, 46–61, and 62–77 are S1 cells cultured for 12 days, 10 days, 5 days, and 3 days respectively. The second dataset contains 3535 malignant T4-2 cells taken from 44 images. Images 1–14, 15–26, 27–36, and 37–44 are T4-2 cells cultured in 5 days, 10 days, 11 days, and 4 days respectively. The third dependent dataset contains both malignant T4-2 and non-neoplastic S1 cells taken from the direct combination of all the 121 images. The time points were selected to span the growth progression of the non-neoplastic cultured cells. Optical sections from 3D images of individual nuclei, showing representative NuMA staining for each of the phenotypes, are displayed in the Methods section.

### Clustering LBF distributions using traditional approaches

Using an automated image analysis method developed earlier [5], we extracted the local bright staining features of NuMA protein and quantified their radial distribution in each nucleus in all the 121 S1 and T4 images. In this way, we obtained 2673 and 3535 LBF distributions for S1 and T4 cells respectively. Each distribution is represented by the normalized density of bright NuMA protein feature as a function of the normalized distance from the perimeter of the nucleus to its center (see Methods for further details).

**Figure 1:**
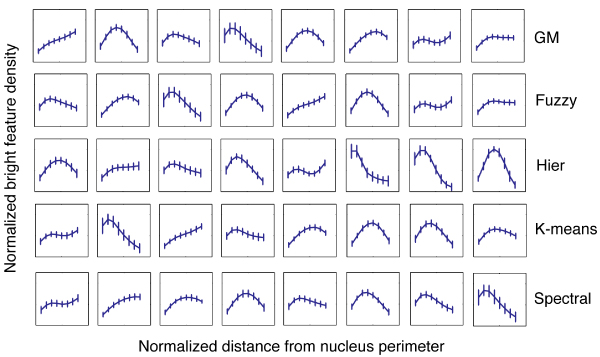
**Clustering 2673 non-neoplastic S1 cells into 8 clusters according to the similarities of their LBF distributions**. Rows from the top to the bottom are the results of Gaussian mixture model clustering with spherical kernel (GM), fuzzy C-means clustering (Fuzzy), hierarchical clustering with complete link (Hier), K-means, and spectral clustering respectively (Spectral). Each cluster is represented by the centroid (curve) and the standard deviation (small vertical bar) of the LBF distributions in the cluster. The horizontal axis of each of the 5 × 8 panels is the normalized distance from the nucleus perimeter, the range being [0,1]. The vertical axis is the normalized bright feature density, the range being [0,2]. Also see Methods for the description of the LBF analysis.

Using traditional approaches of fuzzy C-means clustering, Gaussian mixture model clustering (with a spherical kernel), K-means, hierarchical clustering (with a complete link scheme), and spectral clustering [6-14], we divided the dataset into a number of clusters according to the similarities of their LBF distributions. Figure 1 shows the results for each of these traditional approaches when the dataset of 2673 non-neoplastic S1 cells is divided into 8 clusters. The final result, as we show below, is not dependent on the number of clusters. Each cluster is represented by the centroid (curve) and standard deviation (small vertical bar) of the LBF distributions in the cluster. Clearly, the different methods cluster the data in different ways.

Table 1 shows the consistencies between these clustering results evaluated by pair-wise *F*-measure (see Methods). The results show that quantitatively the consistencies between the clusters produces from each approach are unsatisfactory. For instance, the *F*-measures between the hierarchical clustering and the Gaussian mixture model, fuzzy C-means, K-means, and spectral clustering are 0.5205, 0.5270, 0.4543, and 0.5365 respectively (the fourth row in Table 1). The *F*-measures between the spectral clustering and the Gaussian mixture model, fuzzy C-menas, hierarchical clustering, and K-means are 0.6282, 0.6177, 0.5365, and 0.6253 respectively (the sixth row in Table 1).

**Table 1:** Pair-wise *F*-measures for the clustering results generated by the five traditional clustering approaches, as shown in Figure 1.

	GM	Fuzzy	Hier	Kmeans	Spectral
GM	1.0000	0.8837	0.5205	0.6296	0.6286
Fuzzy	0.8837	1.0000	0.5270	0.6932	0.6177
Hier	0.5205	0.5270	1.0000	0.4543	0.5365
Kmeans	0.6296	0.6932	0.4543	1.0000	0.6253
Spectral	0.6286	0.6177	0.5365	0.6253	1.0000

### Finding consensus LBF clusters using probabilistic ensemble clustering

As shown in Table 1, different clustering methods may generate different results for the same dataset and the agreement between them can be low. This is because each clustering method assumes certain data distributions and cluster characteristics. For instance, the Gaussian mixture model assumes clusters satisfy the Gaussian distribution. K-means works well for clusters of convex shapes. Thus, some algorithms might perform well for specific datasets and not for others. In general, no single clustering method can successfully handle different types of cluster structure. In addition, even different initializations and parameter settings of the same method, for instance, K-means and Gaussian mixture model, may generate different clustering results. As a result, selecting an optimal clustering method is non-trivial or even impossible in many cases. A reasonable way to get a reliable partition of a dataset is to derive a consensus from multiple clustering results, the assumption being that the judgment made by a committee is more robust and unbiased than those made by individuals. This idea, called ensemble clustering, has been investigated in some literatures and several major benefits have been identified [15-21]. First, ensemble-clustering can improve the robustness of clustering. The clusters generated tend to be less sensitive to noise, outliers, initialization, or sampling variations compared to individual clustering methods. Second, ensemble clustering does not need *a priori *information about the number of clusters, but can effectively determine the most probable number of clusters. Third, ensemble clustering can detect outliers. This ability is closely associated with the ability of determining the number of clusters.

Several different ensemble-clustering methods have become available. In [15], a voting algorithm based on hierarchical clustering of the co-association matrix (which represents how often each pair of data appears in the same cluster) is used to derive the consensus clusters. In [16], Strehl and Ghosh developed an evidence accumulation and a hypergraph representation ensemble clustering method. In [17], Topchy et al proposed a mutual-information-based method. In [20], Fischer and Buhmann developed a bootstrap algorithm by first relabeling the data in each clustering result to find the correspondence and then using a voting scheme to find consensus.

In this work, we used a probabilistic ensemble approach based on Bayesian latent variable induction [21-23] (see Methods). Assuming that the clustering results generated by individual methods, i.e., Gaussian mixture model, fuzzy C-means, K-Means, hierarchical clustering, and spectral clustering, are independent of each other, the Bayesian latent variable induction method is able to obtain the statistically optimal combination of individual clustering results as shown by Chickering and Heckerman in [21]. A similar probabilistic ensemble approach has also been adopted by Topchy in [18] where accurate consensus was obtained from unreliable individual clustering results.

**Figure 2:**
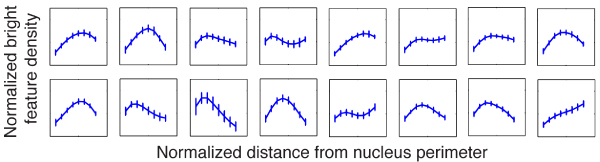
**Consensus clusters of the five clustering results in Figure 1, generated by probabilistic ensemble clustering approach**. The number clusters, i.e., 16, is automatically determined by the algorithm. Like Figure 1, each curve represents the centriod of the cluster. The vertical bar represents the standard variation on the corresponding bin. The horizontal axis of each panel is the normalized distance from nucleus perimeter, the range being [0,1], and the vertical axis is the normalized bright feature density with the range being [0,2].

Using the probabilistic ensemble clustering approach (see Methods for detail), we derived the statistically optimal consensus from different data partition results generated by the five traditional clustering methods mentioned above. Figure 2 shows the result of combining the clusters generated by the five traditional approaches as shown in Figure 1 using the probabilistic ensemble approach. The number of clusters, 16, is automatically determined as a result of finding the consensus.

Table 2 further shows the comparison of our method with traditional methods in terms of the number of clusters predefined in individual clustering methods (the second row) and those automatically determined by the probabilistic ensemble clustering approach (the third row) for the dataset containing both S1 and T4-2 cells. Clearly, the number of clusters automatically determined by the probabilistic ensemble approach does not vary significantly with the number of clusters predefined for individual clustering methods. When the number of clusters predefined changes from 8 to 26, the number of clusters identified by the probabilistic ensemble clustering approach is much more stable, ranging from 19 to 25.

**Table 2:** Number of clusters (the second row) predefined in the individual clustering methods (i.e., Gaussian mixture model, fuzzy C-means, hierarchical clustering, K-means and spectral clustering) and those automatically determined by the probabilistic ensemble clustering method for both S1 and T4-2 cells (the third row).

Methods	Number of Clusters											
Traditional methods	4	6	8	10	12	14	16	18	20	22	24	26
Probabilstic ensemble-clustering	19	18	18	16	19	20	19	20	22	22	23	25

### Computing cluster histograms

**Figure 3:**
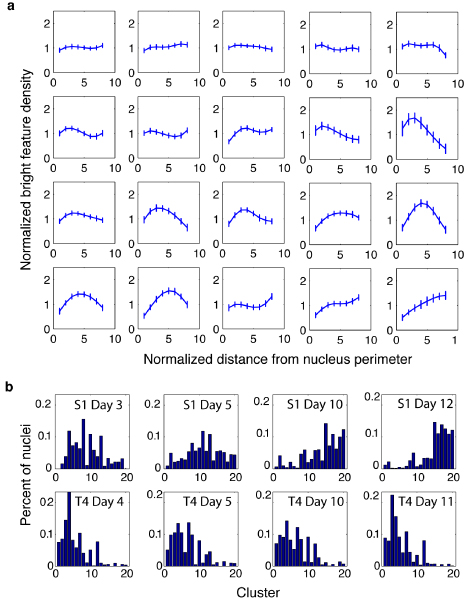
**LBF distribution clusters and cluster histograms for 6208 S1 and T4-2 cells cultured for different numbers of days**. (a) Twenty LBF distribution clusters automatically determined by probabilistic ensemble clustering of the results generated by Gaussian mixture model, fuzzy C-means, hierarchical clustering, K-means, and spectral clustering. The number of the clusters predefined for these baseline methods is 14. The clusters are ordered from the left to the right and the top to the bottom according to their peak locations. (b) From the left to right and the top to the bottom: cluster histograms of non-neoplastic S1 cells cultured in 3 days, 5 days, 10 days, and 12 days, and of malignant T4-2 cells cultured in 4 days, 5 days, 10 days, and 11 days.

With clusters reliably determined, we then calculated the number of LBF distributions falling into each cluster for each of the 8 populations of cells, i.e., non-neoplastic S1 cells cultured for 3 days, 5 days, 10 days, and 12 days, as well as malignant T4-2 cells cultured for 4 days, 5 days, 10 days, and 11 days. By doing so, we obtained a cluster histogram for each of the 8 populations of cells. Figure 3a shows the 20 clusters automatically determined by combining the clustering results of Gaussian mixture model, fuzzy C-means, hierarchical clustering, K-means, and spectral clustering using the probabilistic ensemble clustering for the dataset containing 2673 non-neoplastic S1 cells and 3535 malignant T4-2 cells. The number of the clusters predefined for these baseline methods is 14 (as shown in Table 2). In fact, the cluster histograms and the phenotype trees built in later step are insensitive to the number of clusters predefined for traditional clustering methods as will be shown in the Methods section. The 20 clusters in Figure 3a are ordered from the left to the right and the top to the bottom according to their peak locations. The first 8 clusters are approximately flat. In the 9^th ^to the 20^th ^clusters the peak location shifts from the left to the right. Figure 3b shows the cluster histograms for the 8 populations of cells. For S1 cells, the cluster histograms (the top row in Figure 3b) are remarkably different between the early stage (e.g. S1 Day 3) and the completion of acinar morphogenesis (e.g., S1 Day 12). The peak of the histogram gradually shifts from the left to the right as the number of days in culture increases, indicating a gradual modification during the 12-day *in vitro *morphogenesis process. This is consistent with the fact that NuMA staining is diffusely distributed within the nuclei of proliferating cells, but aggregates into foci of increasing size as cells arrest proliferation and complete acinar morphogenesis. Therefore, the cluster histograms statistically reflect the phenotype of non-neoplastic S1 cells. Moreover, the peak of the histogram profile does not change significantly for malignant T4-2 cells cultured for different numbers of days (bottom row in Figure 3b). This is also consistent with the fact that NuMA staining is diffusely distributed within T4-2 nuclei despite the number of days in culture. Interestingly, the cluster histograms of malignant T4-2 cells differ significantly from those of non-neoplastic S1 cells. The consistency of cluster histograms and cell types indicates that it is meaningful to develop a method to predict cell phenotypes and their sub-categories based on cluster histograms.

### Constructing phenotype trees

**Figure 4:**
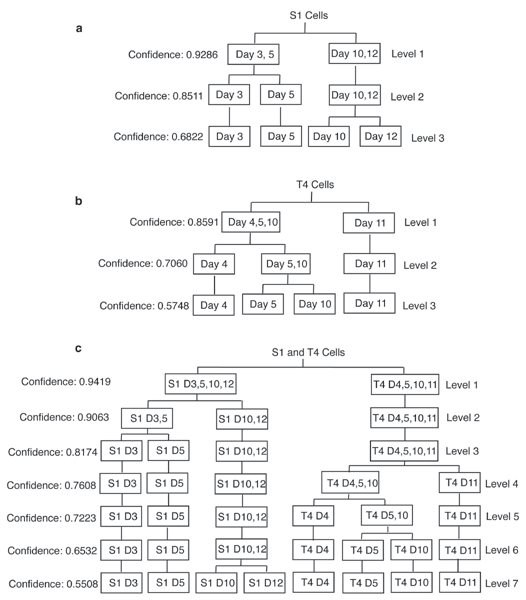
**Phenotype trees constructed for (a) non-neoplastic S1 cells, (b) malignant T4-2 cells, and (c) both S1 and T4-2 cells cultured for a different number of days**. The certainty of hierarchically grouping the cells of the predefined phenotypes (indicated by the leaf nodes in the highest level of the tree) into statistically more significant groups of the phenotypes is indicated by the *confidence *values at each level of the tree.

Using the approach introduced in the Methods section, we have constructed phenotype trees to show how the phenotypes, defined by the behavior of the cells in 3D culture, can be hierarchically grouped and the statistical significance of each grouping calculated. Figure 4a shows the phenotype tree built for non-neoplastic S1 cells. At the first level in this figure, the four phenotypes of S1 cells were divided into two groups. Of the multiple ways to create two groups from four phenotypes, our method found that having S1 cells at day 12 and day 10 in one group and S1 cells at day 3 and day 5 in the other resulted in the highest confidence value, of 0.9286 (Figure 4a). In the second level of the tree, our method divided S1 cells into three phenotype groups. The results showed that having S1 cells at day 12 and day 10 as one group, S1 cells at day 5 as the second group, and S1 cells at day 3 as the third provided the highest confidence value of 0.8511. This was lower than the confidence of dividing S1 cells into two groups. Finally, the method divided S1 cells into four groups which resulted in a confidence value of 0.6822 (Figure 4a). This phenotype tree indicates we can distinguish S1 cells at day 3 and 5 from those cultured at day 10 and 12 days with high confidence.

Using the same approach, we constructed the phenotype trees for malignant T4-2 cells and for the combination of S1 and T4-2 cells, as shown in Figure 4b and Figure 4c respectively. Figure 4b shows that we can distinguish T4-2 cells cultured at day 4, day 5, day 10 from those cultured at day 11 in relatively high confidence (0.8591; the first level of Figure 4b). However, if we want to distinguish T4-2 cells cultured for different numbers of days, the confidence drops to 0.5748. Figure 4c shows that we can distinguish S1 and T4-2 cells with very high confidence (0.9419; see the first level of Figure 4c). However, the confidence drops as level increases. The certainty in distinguishing all the 8 phenotypes drops to 0.5508 at the highest level of the tree. In general, the phenotype trees provide us a way to evaluate how the phenotypes, defined by the behavior of the cells in 3D culture, can be hierarchically grouped and the statistical significance between each grouping calculated.

## Discussion and conclusions

We have developed a cluster analysis approach that can robustly link any given set of multivariate features measured on a per cell basis to the phenotype of the cells as defined by their macroscopic biology. The technique uses a probabilistic ensemble approach to group the measured multivariate features into a set of consensus clusters. This method provides a novel way of linking the phenotypes of groups of cells to cluster histograms that describe the distribution of the measured features across the consensus clusters. Then, by forming various groupings of the cluster histograms, the technique permits the formation of a phenotype tree and calculations of the statistical significance between each of the groups. If two groups of cells are found to be significantly different, one can conclude that the features measured in the cells can distinguish the groups that are indeed different. If the two groups are not significantly different, one can only conclude that the measured feature does not change between these groups. It does not imply that that the groups are necessarily identical.

**Figure 5:**
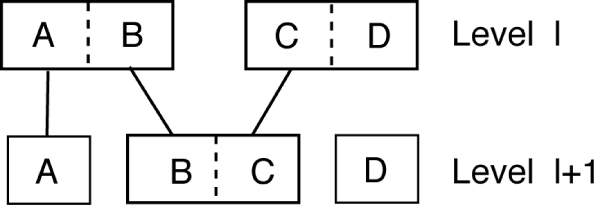
**Illustration of the inconsistent phenotype grouping between successive levels**. Each solid rectangle represents a phenotype node. A dashed line indicates combination operation. Phenotype groupings at level l and l+1 are inconsistent as the node BC at level l+1 is formed by breaking node AB and node CD at level l into two parts and combining one part of each node. In this case, the hierarchical structure cannot be represented as a tree.

The phenotype tree is a hierarchical representation of the possible grouping of the defined cell phenotypes. As such, a node in the tree at level *l *can be spitted into at most two nodes at level *l*+1. However, the method used in building the tree does not prevent inconsistent group divisions between level *l *and *l*+1. Thus a node at level *l*+1 can be a combination of two partial nodes at level *l*, as shown in Figure 5. As a result, the hierarchical structure cannot be represented as a tree. To solve the problem, we can add a consistency constrain to make the phenotype groups, between different tree levels, coherent. Alternatively, we can use directed acyclic graphs (DAG) to represent the hierarchical structure of cell phenotype without adding any consistency constrain.

We have shown how the cluster analysis technique can be applied to the radial LBF distributions of a chromatin-associated protein, NuMA [24], measured on a per cell basis from non-neoplastic S1 and malignant T4-2 HMECs, cultured in a 3D environment for up to 12 days. The results showed, that for this measured feature, the method can distinguish the non-neoplastic S1 cells and malignant T4-2 cells with 94.19% accuracy, and proliferating S1 cells from S1 cells differentiated into acinar structures with 92.86% accuracy. The phenotype tree also shows that the method only distinguishes the four phenotypes of S1 cells with 68.22% accuracy. However, when the two phenotypes S1-day 10 and S1-day 12 are considered as one group, the ability to distinguish that group from S1-day 5 and S1-day 3 jumps to 85.11%. This result demonstrates the power of the phenotype tree, which in this case shows that the distribution of NuMA changes moderately between the phenotypes S1-day3 and S1-day 5, markedly between the phenotypes S1-day 5 and S1-day 10 but then does not changed significantly in S1 cells at 10 days compared to 12 days in culture. These results correlate with the behavior of cultured S1 cells and clearly show that the reorganization of NuMA that occurs during the morphogenic process of these cells is almost complete at 10 days of culture. In other words, S1-day 10 and S1-day 12 are not significantly different phenotypes, based on NuMA distribution. These results are echoed by the cluster histograms for the S1 cells. Clearly marked differences are seen between cluster histograms of the phenotypes S1-day 5 and S1-day 10 and not between the phenotypes S1-day 10 and S1-day 12. Further, the method only distinguishes the four phenotypes of T4-2 cells with 57.48% accuracy. This result also correlates with the behavior of these malignant cells that continue to proliferate throughout the 12 day culture period. This result simply demonstrates that based on NuMA distribution, the phenotypes T4-2-day 4, T4-2-day 5, T4-2-day 10 and T4-2-day 11 are not significantly different. It does not rule out the possibility that introducing other measured features could reveal differences between such phenotypes.

Collectively our data demonstrate the quantitative ability of clustering-based analysis to link microscopically measurable features with the behavior of the cells. The methods described demonstrate that it is possible to distinguish populations of cells based on the nuclear organization of a chromatin-associated protein, NuMA. This work paves the way for our longer term goal of producing a method capable of turning high resolution fluorescence images of human mammary epithelial tissue into tissue-maps that report the probable non-neoplastic, premalignant and malignant phenotype at cellular resolution.

## Methods

**Figure 6:**
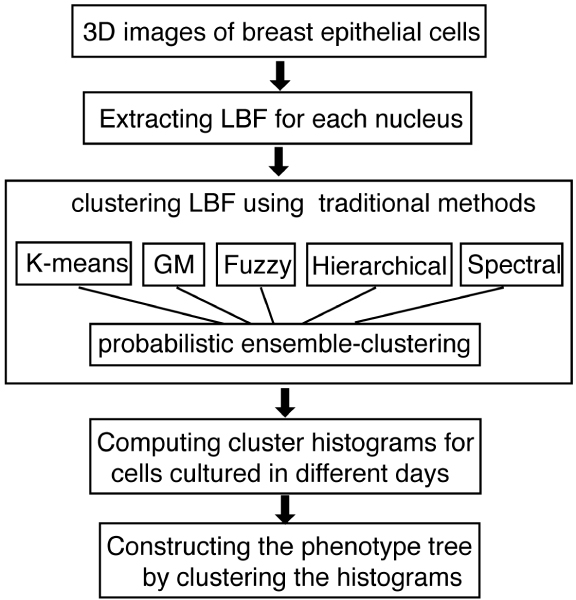
**Diagram of the phenotype clustering algorithm**. Details of the image acquisition and the extraction of the LBF for each nucleus is described in [5].

Our phenotype clustering approach contains four steps (Figure 6). Firstly, we used a previously developed image analysis method [5] to analyze each fluorescence image acquired by the Zeiss 410 3D confocal microscope, and obtained LBF distributions for all nuclei within many images. Secondly, we grouped thousands of nuclei into clusters based on the similarities between their LBF distributions. For this purpose, we tested K-means clustering, fuzzy C-means clustering, Gaussian mixture model, spectral clustering, and hierarchical clustering methods [6-14] and found that the consistency between the different clustering results, evaluated by an *F*-measure, were relatively low. Because it is difficult to choose the best approach, we developed a probabilistic ensemble approach based on Bayesian latent variable induction to combine the different clustering results into a set of consensus clusters of LBF distributions. We then analyzed how nuclei were distributed across the consensus clusters, and obtained a cluster histogram for cells of each defined phenotype. Finally, we constructed hierarchical phenotype trees to show how the predefined phenotypes could be hierarchically grouped and the statistical significance of each grouping calculated. The trees were structured so that nodes at lower levels correspond to phenotype groups with larger statistical difference.

### Extracting LBF distributions from nuclei

**Figure 7:**
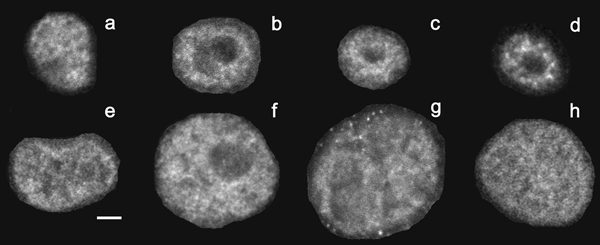
**Fluorescence micrographs showing representative NuMA staining patterns in individual nuclei for eight different phenotypes**. In previous work [5] the radial nuclear distribution of NuMA was analyzed from 3D multichannel fluorescence images of thousands of individual nuclei. The human mammary epithelial cells were either non-neoplastic (top row) or malignant (bottom row) and were cultured in Matrigel™ (3D culture) for up to 12 days. Optical sections from 3D images, taken through the approximate midplane of individual nuclei are displayed. The optical sections were chosen to show representative features of the NuMA staining pattern. Panels a, b, c and d, show NuMA staining from non-neoplastic cells cultured for 3, 5, 10 and 12 days, representing cells present in incremental differentiation steps, respectively. Panels e, f, g, and h, show NuMA staining from malignant cells cultured for 4, 5, 10 and 11 days, representing cells present in tumors of increasing sizes, respectively. Notice that the nuclei of malignant cells are consistently larger than the nuclei of non-neoplastic cells. The bar represents 5 microns.

Using Zeiss 410 confocal laser-scanning microscope with planapochromatic 63×, 1.4 numerical aperture lens, we acquired hundreds of 3D images of non-neoplastic S1 and malignant T4-2 cells cultured for up to 12 days. Figure 7 shows optical sections from the middle of 3D images of individual nuclei, showing representative NuMA staining for each of the phenotypes described in this work.

**Figure 8:**
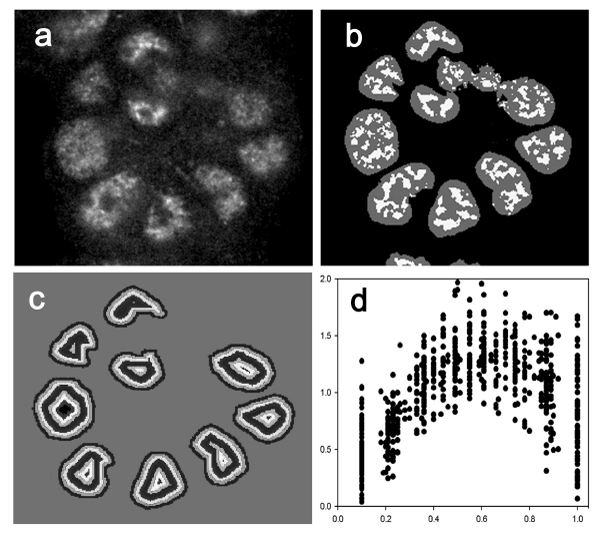
**LBF analysis of the distribution of NuMA from 3D images**. (a) Fluorescence micrograph of Texas red-immunolabeled NuMA from a single optical section, in differentiated non-neoplastic S1 cells. (b) The corresponding processed image section showing a composite view of the detected local bright features (light gray) of NuMA, extracted by the local bright feature analysis overlaid on the nuclear segmentation mask (dark gray). (c) Concentric terraces resulting from the application of the distance transform on the segmentation mask, which allows the radial distribution of NuMA to be calculated. (d) A set of LBF distribution profiles of NuMA calculated from differentiated non-neoplastic S1 cells. The relative density of NuMA bright features (ordinate) is plotted as a function of the relative distance from the perimeter (0.0) to the center (1.0) of the nuclei (abscissa).

In an earlier study, an image analysis method was developed to extract the local bright staining features of NuMA protein and quantify their radial distribution in each individual nucleus ([5], also see Figure 8). The technique first used a model-based method to automatically segment individual nuclei in the DAPI-stained channel of the confocal images. It then divided the brightness at each point within a nucleus by the local average brightness in a region surrounding that point in the NuMA-stained channel, thus isolating the local brightness features (LBF) of each nucleus. Then, the radial distribution of these bright features was computed using a distance transform. The transform calculates the shortest distance of each point within a nucleus to the nuclear boundary and in doing so, divides each nucleus into a set of concentric terraces of equal thickness. In each terrace, the density of local bright features was calculated as the number of bright pixels divided by the total number of pixels. To account for variations in the number of terraces per nucleus due to variations in nucleus size and shape, the density per terrace was normalized so that the average density of bright features was 1 for each nucleus, and the distances from nuclear perimeter were also normalized to the range of [0, 1.0]. Through the above process, a radial distribution of LBF was derived for each nucleus, represented by the normalized density of bright features as a function of the normalized distance from the perimeter of the nucleus to its center.

### Clustering LBF distributions using traditional approaches

Our phenotype clustering algorithm is based on the radial distribution of LBFs. To group the LBF distribution of thousands of nuclei into clusters of similar patterns, we first tested traditional clustering approaches, including the most widely used K-means, fuzzy C-means clustering, Gaussian mixture model (with a spherical kernel), hierarchical clustering (with the complete link scheme), and the spectral clustering methods [6-14].

Since different clustering methods generate different clusters, we computed the pair-wise *F*-measure score to evaluate the consistencies between different clustering results. The *F*-measure is defined as follows. For any two data partition *U *and *V*, denote the *i*th cluster in partition U as *u*_*i*_, and the *j*th cluster in partition V as *v*_*j*_. The proportion of data in *u*_*i *_that is also in *v*_*j *_is *R *= |*u*_*i *_⋂ *v*_*j*_|/|*u*_*i*_|, and the portion of data in *v*_*j *_that is also in *u*_*i *_is *P *= |*u*_*i *_⋂ *v*_*j*_|/|*v*_*j*_|. Define *F*(*i*, *j*) = 2*PR*/(*P*+*R*). The score to measure the consistency of the partition *V *with partition *U *is *F*_0 _= [Σ|*u*_*i*_|*max*_*j*_F(*i*, *j*)]/[Σ|*u*_*i*_|], where |*u*_*i*_| is the number of data point in *u*_*i*_. To make it symmetrical, the final *F*-measure is defined as *F *= (*F*_0_+*F*_0_')/2, where *F*_0_' denotes the transpose of *F*_0_.

### Probabilistic ensemble clustering

The probabilistic ensemble clustering approach we used to derive the consensus clusters from multiple clustering results is based on general Bayesian latent variable induction [21-23]. Let us suppose we have *M *different clustering approaches, generating *M *data partition *C*_*i *_(*i *= 0,..., *M*) of the same dataset *D *containing *N *data points. Our purpose is to infer the optimal consensus data partition *L *from the multiple partitions *C*_*i*_. We notice that one simple yet reasonable assumption is that we can treat all the *M *clustering results *C*_1_,..., *C*_*M *_as independent samples drawn from the same underlying distribution *L*. In another words, we can assume that the distributions of *C*_1_,..., *C*_*M *_are conditionally independent of each other given the latent variable *L*. This assumption allows us consider the following Bayesian latent variable induction model.

Let us suppose the *i*th clustering approach divides the dataset into *r*_*i *_clusters, then each *C*_*i *_has *r*_*i *_states (categorical labels), i.e., 1,..., *r*_*i*_. Initially the consensus *L *may divide the dataset into *k *clusters (the final value k* is automatically determined; see below), then *L *has *k *states, i.e., 1,..., *k*. Since each LBF distribution vector in the dataset is assigned a cluster label by *C*_*i*_, it takes a specific state value on *C*_*i*_. Denote *s *= (*C*_1 _= *c*_1_, *C*_2 _= *c*_2_,...., *C*_*M *_= *c*_*M*_), where *c*_*i *_(*i *∈ [0, *M*]) takes one state in 1,..., *r*_*i*_.

Upon initialization of the latent variable *L*, we randomly assign each of the *N *data points one of the *k *states. Given a data *s *which is assigned state label *c*_*i *_by the *i*th clustering method *C*_*i*_, we derive its probability of taking state label *l *(where *l *∈ [1, *k*]) in consensus *L*, i.e., P(*L *= *l*|*s*). Based on the conditional independence assumption, we have

P(L(j)=l|s(j))∝P(s(j)|L(j)=l)=∏i=1MP(Ci(j)=ci|L(j)=l)
 MathType@MTEF@5@5@+=feaafiart1ev1aaatCvAUfKttLearuWrP9MDH5MBPbIqV92AaeXatLxBI9gBaebbnrfifHhDYfgasaacH8akY=wiFfYdH8Gipec8Eeeu0xXdbba9frFj0=OqFfea0dXdd9vqai=hGuQ8kuc9pgc9s8qqaq=dirpe0xb9q8qiLsFr0=vr0=vr0dc8meaabaqaciaacaGaaeqabaqabeGadaaakeaacqWGqbaucqGGOaakcqWGmbatdaahaaWcbeqaaiabcIcaOiabdQgaQjabcMcaPaaakiabg2da9iabdYgaSjabcYha8jabdohaZnaaCaaaleqabaGaeiikaGIaemOAaOMaeiykaKcaaOGaeiykaKIaeyyhIuRaemiuaaLaeiikaGIaem4Cam3aaWbaaSqabeaacqGGOaakcqWGQbGAcqGGPaqkaaGccqGG8baFcqWGmbatdaahaaWcbeqaaiabcIcaOiabdQgaQjabcMcaPaaakiabg2da9iabdYgaSjabcMcaPiabg2da9maarahabaGaemiuaaLaeiikaGIaem4qam0aa0baaSqaaiabdMgaPbqaaiabcIcaOiabdQgaQjabcMcaPaaakiabg2da9iabdogaJnaaBaaaleaacqWGPbqAaeqaaOGaeiiFaWNaemitaW0aaWbaaSqabeaacqGGOaakcqWGQbGAcqGGPaqkaaGccqGH9aqpcqWGSbaBcqGGPaqkaSqaaiabdMgaPjabg2da9iabigdaXaqaaiabd2eanbqdcqGHpis1aaaa@6A52@

where *j *denotes the *j*th data in the dataset *D*, *P*(*C*_*i *_= *c*_*i*_|*L *= *l*) (*i *∈ [0, *M*]) can be easily obtained by counting and normalizing the occurrence frequency of data that are assigned the state label *c*_*i *_by the clustering method *C*_*i*_, given the data is assigned the state label *l *in *L*. Once P(*L *= *l*|*s*) is available, we use it to resample and update the state label of each data in *L*. The above process repeats until all the data do not change states. This will lead to the estimation of an optimal consensus function *L *for a specified number of clusters, *k*.

We observe that when the data samples (LBFs) are independent of each other, the likelihood of the latent variable *L *which has *k *states can be estimated as

P(k|D)=∏j=1NP(k|s(j))=∏j=1N∑l=1kP(k,L(j)=l|s(j))
 MathType@MTEF@5@5@+=feaafiart1ev1aaatCvAUfKttLearuWrP9MDH5MBPbIqV92AaeXatLxBI9gBaebbnrfifHhDYfgasaacH8akY=wiFfYdH8Gipec8Eeeu0xXdbba9frFj0=OqFfea0dXdd9vqai=hGuQ8kuc9pgc9s8qqaq=dirpe0xb9q8qiLsFr0=vr0=vr0dc8meaabaqaciaacaGaaeqabaqabeGadaaakeaacqWGqbaucqGGOaakcqWGRbWAcqGG8baFcqWGebarcqGGPaqkcqGH9aqpdaqeWbqaaiabdcfaqjabcIcaOiabdUgaRjabcYha8jabdohaZnaaCaaaleqabaGaeiikaGIaemOAaOMaeiykaKcaaOGaeiykaKcaleaacqWGQbGAcqGH9aqpcqaIXaqmaeaacqWGobGta0Gaey4dIunakiabg2da9maarahabaWaaabCaeaacqWGqbaucqGGOaakcqWGRbWAcqGGSaalcqWGmbatdaahaaWcbeqaaiabcIcaOiabdQgaQjabcMcaPaaakiabg2da9iabdYgaSjabcYha8jabdohaZnaaCaaaleqabaGaeiikaGIaemOAaOMaeiykaKcaaOGaeiykaKcaleaacqWGSbaBcqGH9aqpcqaIXaqmaeaacqWGRbWAa0GaeyyeIuoaaSqaaiabdQgaQjabg2da9iabigdaXaqaaiabd6eaobqdcqGHpis1aaaa@6663@

It is apparent that we can maximize the likelihood in Eq. (2) to find the best *k *over a specified range. In practice, we can often avoid iteration in Eq. (2) by directly assigning a big *k*. After convergence in solving Eq. (1), there are k* (*k *≥ k*) states in *L *that have non-zero number of data points. This k* value is the statistically optimal *k *value automatically determined.

### Computing cluster histograms for cells of different phenotypes

Once we obtained reliable clusters of LBF distributions of individual nuclei, we analyzed how the cells belonging to different phenotypes, defined by the behavior of the cells, (i.e., S1 and T4-2 cells cultured in different days) were distributed across the various LBF clusters. For this purpose, we counted the number of nuclei whose LBF distribution fell into each cluster for each phenotype, i.e., S1 cells cultured for 3, 5, 10, and 12 days, and T4-2 cells cultured for 4, 5, 11, and 12 days. By doing so, we obtained the cluster histogram of each phenotype, represented by the percentile of nuclei as a function of clusters. The cluster histograms do not only directly link to predefined phenotypes (as shown in Figure 3) but also provided more detail information compared to cell malignancy and days in culture.

### Constructing the phenotype tree

**Figure 9:**
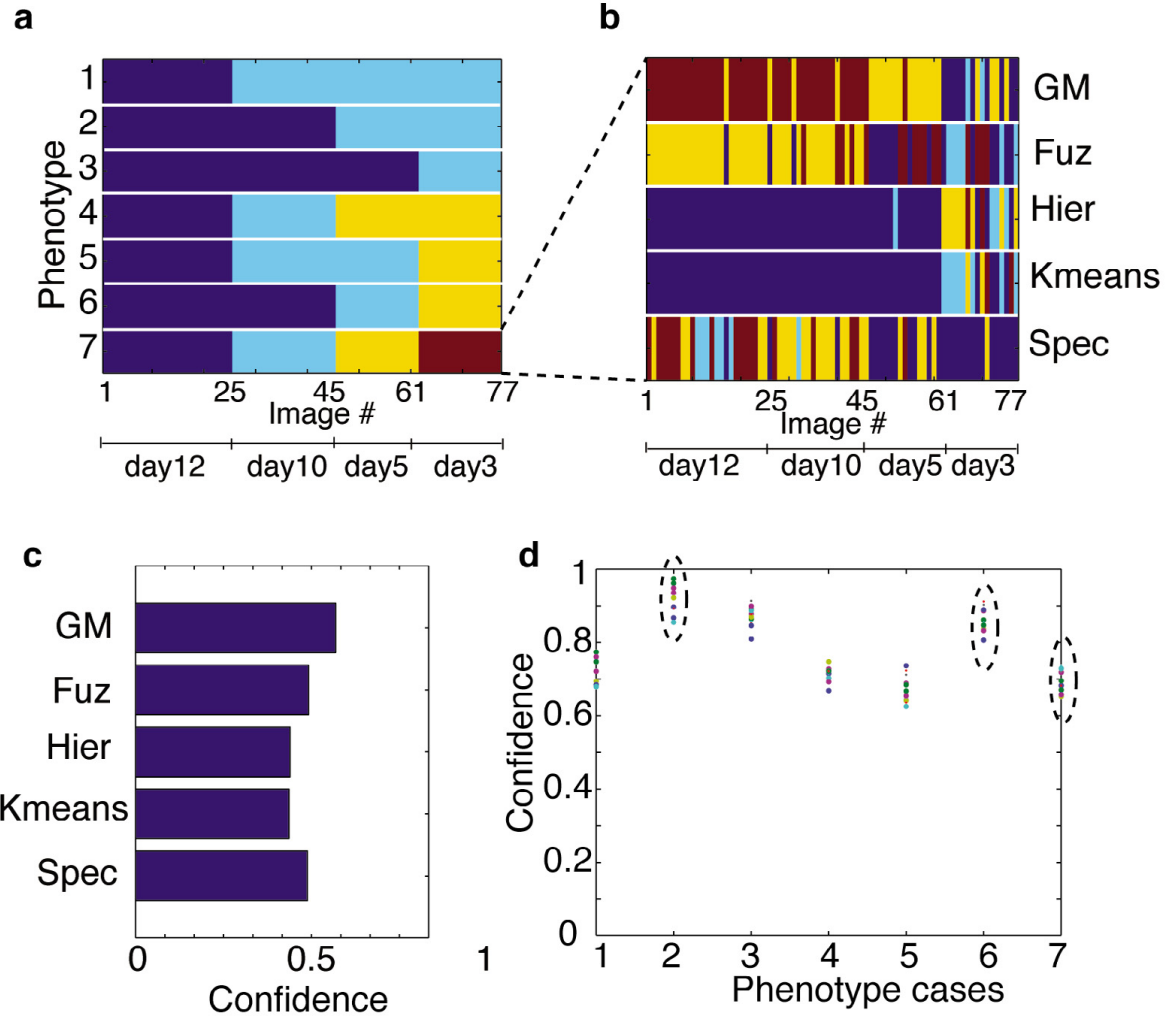
**An illustration of phenotype tree construction process**. (a) Images 1–25, 26–45, 46–61, and 62–77 correspond to non-neoplastic S1 cells cultured for 12 days, 10 days, 5 days, and 3 days respectively. There are 7 possible ways of grouping the phenotypes. Each row corresponds to one possible way. Different colors represent different phenotype groups. The first 3 rows correspond to grouping the 4 predefined phenotypes into 2 groups. The next 3 rows correspond to grouping the phenotypes into 3 groups, and the last row correspond to 4 groups. (b) Taking the 4 phenotype group case (last row in (a)) as an example, we used traditional clustering methods to divide the cluster histogram of the image (one cluster histogram per image) into the same number of clusters (i.e., 4 in this example). Each row corresponds to the clustering result of one method. (c) The *F*-measures computed by pairing the phenotype group in the last row of (a) with each clustering result in (b). The maximum *F*-score, which in this case is achieved by the Gaussian Mixture Model approach (GM), is selected as the *confidence *of the corresponding cell phenotype grouping. (d) Confidence values as functions of different cases of phenotype groupings. We tested the confidence values under different number of clusters predefined for clustering LBF distributions using the five traditional methods (i.e., the second step of our algorithm, see Figure 6) as shown by dots of different colors. The numbers of clusters we tested were 4 to 26 with step size of 2. The consistent distribution of the dots indicates that our phenotype tree construction method is insensitive to the number of clusters we selected for clustering LBF distributions.

Taking the non-neoplastic S1 cells cultured for different days as an example, our method in constructing the tree is as follows. For all the *N *images of S1 cells, we assume images of the same day are of the same phenotype and morphogenesis progresses montotonically, as defined by biologists. This allowed us to group the images sequentially, leading to ∑i=1P−1CP−1i
 MathType@MTEF@5@5@+=feaafiart1ev1aaatCvAUfKttLearuWrP9MDH5MBPbIqV92AaeXatLxBI9gBaebbnrfifHhDYfgasaacH8akY=wiFfYdH8Gipec8Eeeu0xXdbba9frFj0=OqFfea0dXdd9vqai=hGuQ8kuc9pgc9s8qqaq=dirpe0xb9q8qiLsFr0=vr0=vr0dc8meaabaqaciaacaGaaeqabaqabeGadaaakeaadaaeWaqaaiabdoeadnaaDaaaleaacqWGqbaucqGHsislcqaIXaqmaeaacqWGPbqAaaaabaGaemyAaKMaeyypa0JaeGymaedabaGaemiuaaLaeyOeI0IaeGymaedaniabggHiLdaaaa@3A97@ possible ways of grouping the different phenotypes, where *C *denotes the combination operation and *P *is the number of defined cell phenotypes. For instance, if *P *= 4, then the total number of possible ways of grouping phenotypes is 7 (i.e., ∑i=13C3i
 MathType@MTEF@5@5@+=feaafiart1ev1aaatCvAUfKttLearuWrP9MDH5MBPbIqV92AaeXatLxBI9gBaebbnrfifHhDYfgasaacH8akY=wiFfYdH8Gipec8Eeeu0xXdbba9frFj0=OqFfea0dXdd9vqai=hGuQ8kuc9pgc9s8qqaq=dirpe0xb9q8qiLsFr0=vr0=vr0dc8meaabaqaciaacaGaaeqabaqabeGadaaakeaadaaeWaqaaiabdoeadnaaDaaaleaacqaIZaWmaeaacqWGPbqAaaaabaGaemyAaKMaeyypa0JaeGymaedabaGaeG4mamdaniabggHiLdaaaa@3673@). Among these 7 cases, 3 cases (i.e., C31
 MathType@MTEF@5@5@+=feaafiart1ev1aaatCvAUfKttLearuWrP9MDH5MBPbIqV92AaeXatLxBI9gBaebbnrfifHhDYfgasaacH8akY=wiFfYdH8Gipec8Eeeu0xXdbba9frFj0=OqFfea0dXdd9vqai=hGuQ8kuc9pgc9s8qqaq=dirpe0xb9q8qiLsFr0=vr0=vr0dc8meaabaqaciaacaGaaeqabaqabeGadaaakeaacqWGdbWqdaqhaaWcbaGaeG4mamdabaGaeGymaedaaaaa@2FCC@) correspond to grouping the four macroscopically defined phenotypes into 2 groups, 3 cases (i.e., C32
 MathType@MTEF@5@5@+=feaafiart1ev1aaatCvAUfKttLearuWrP9MDH5MBPbIqV92AaeXatLxBI9gBaebbnrfifHhDYfgasaacH8akY=wiFfYdH8Gipec8Eeeu0xXdbba9frFj0=OqFfea0dXdd9vqai=hGuQ8kuc9pgc9s8qqaq=dirpe0xb9q8qiLsFr0=vr0=vr0dc8meaabaqaciaacaGaaeqabaqabeGadaaakeaacqWGdbWqdaqhaaWcbaGaeG4mamdabaGaeGOmaidaaaaa@2FCE@) correspond to grouping them into 3 groups, and 1 case (i.e., C33
 MathType@MTEF@5@5@+=feaafiart1ev1aaatCvAUfKttLearuWrP9MDH5MBPbIqV92AaeXatLxBI9gBaebbnrfifHhDYfgasaacH8akY=wiFfYdH8Gipec8Eeeu0xXdbba9frFj0=OqFfea0dXdd9vqai=hGuQ8kuc9pgc9s8qqaq=dirpe0xb9q8qiLsFr0=vr0=vr0dc8meaabaqaciaacaGaaeqabaqabeGadaaakeaacqWGdbWqdaqhaaWcbaGaeG4mamdabaGaeG4mamdaaaaa@2FD0@) corresponds to grouping them into 4 groups. These 7 cases are shown in Figure 9a. Different colors in each row represent different groups. The first three bins correspond to dividing the S1 cells cultured for 3 days, 5 days, 10 days and 12 days into 2 groups, the next three bins correspond to dividing the cells into 3 groups, and the 7^th ^bin corresponds to dividing the cells into 4 groups.

Our next step is to determine the likelihood of these potential groupings. Assume we want to divide the predefined phenotypes into *p *groups (where *p *= 2,3,4 in the above example). We then grouped the cluster histogram of the 77 S1 cell images into the same number of clusters. To improve reliability we again used multiple clustering algorithms, including K-means, fuzzy C-means clustering, hierarchical clustering, Gaussian Mixture model, and spectral clustering, as used in generating the LBF clusters (see Figure 9b). We then paired each clustering result with the phenotype grouping under consideration, and calculated the degree of agreement between them using the *F*-measure. We then selected the maximum *F*-score as the *confidence *of the corresponding cell phenotype grouping (see Figure 9c). By repeating the process for each potential phenotype grouping, we finally obtained the value of the confidence as the function of the different cases of phenotype grouping.

To further test the sensitivity of this method to the number of clusters predefined when generating the clusters of LBF distributions using the five traditional clustering approaches, we repeated the process for different numbers of clusters predefined for the traditional methods and obtained a set of confidence values for each phenotype grouping case as indicated by the colored dots in each bin of Figure 9d. The result exhibits a central tendency, indicating that the method is insensitive to the number of clusters predefined in clustering the LBF distributions. We then took the median of the confidence values obtained under different number of clusters on each bin as the overall confidence value of the corresponding phenotype grouping.

Given *p*, the number of groups that the predefined phenotype should be grouped into, we selected from all the phenotype grouping cases that have the same number of groups the one that has the maximum confidence value, as the most likely phenotype grouping case under the given *p*. For instance, if we want to group the predefined phenotypes into 2 groups, i.e., *p *= 2, there are three phenotype grouping cases, corresponding to the first three bins in Figure 9d and the first three rows in Figure 9a. The second case has the maximum confidence value (indicated by the left-most dashed ellipse in Figure 9d, which corresponds to the second row of Figure 9a) and is thus taken as the right way of grouping the predefined phenotypes into 2 groups. This means that S1 cells cultured for 10 and 12 days (i.e., images 1–45) belong to one group, and those cultured for 3 and 5 days belong to another (i.e., images 46–77). Using this approach, we determined the most likely phenotype grouping for *p *= 3 and *p *= 4, which correspond to the 6^th ^and 7^th ^bin in Figure 9d and the 6^th ^and 7^th ^row in Figure 9a respectively. These three phenotype groupings constitute the first to the third level of the phenotype tree as shown in Figure 4a.

## Competing interests

The authors declare that they have no competing interests.

## References

[B1] Zink D, Fischer AH, Nickerson JA (2004). Nuclear structure in cancer cells. Nat Rev Cancer.

[B2] Lelièvre SA, Bissell MJ, Pujuguet P (2000). Cell nucleus in context. Crit Rev Eukaryot Gene Expr.

[B3] Dillon N, Festenstein R (2002). Unravelling heterochromatin: competition between positive and negative factors regulates accessibility. Trends Genet.

[B4] Lelièvre SA, Weaver VM, Nickersondagger JA, Larabell CA, Bhaumik A, Petersen OW, Bissell MJ (1998). Tissue phenotype depends on reciprocal interactions between the extracellular matrix and the structural organization of the nucleus. Proc Natl Acad Sci USA.

[B5] Knowles DW, Sudar D, Carol Bator-Kelly, Bissell MJ, Lelièvre SA (2006). Automated local bright feature image analysis of nuclear protein distribution identifies changes in tissue phenotype. Proc Natl Acad Sci USA.

[B6] Dunn JC (1973). A fuzzy relative of the ISODATA process and its use in detecting compact well-separated clusters. Journal of Cybernetics.

[B7] Bezdek JC (1981). Pattern Recognition with Fuzzy Objective Function Algoritms.

[B8] McLachlan G, Basford K (1988). Mixture models: inference and application to clustering.

[B9] Roberts S, Husmeier D, Rezek I, Penny W (1998). Bayesian approaches to Gaussian mixture modeling. IEEE trans Pattern Analysis and Machine Intelligence.

[B10] Jain AK, Murty MN, Flynn PJ (1999). Data clustering: a review. ACM Computing Surveys.

[B11] Hartigan J (1975). Clustering Algorithms.

[B12] Kaufman L, Rousseeuw PJ (1990). Finding groups in data: an introduction to cluster analysis.

[B13] Wu Z, Leahy R (1993). An optimal graph theoretic approach to data clustering: theory and its application to image segmentation. IEEE Tran Pattern Analysis and Machine Intelligence.

[B14] Peng H, He X, Long F (2004). Automatic content extraction of filled form images based on clustering component block projection vectors. Proc IS&T/SPIE 16th Annual Symp of Electronic Imaging, Conf on Document Recognition and Retrieval XI, San Jose, CA, USA.

[B15] Fred A, Jain AK (2002). Evidence: accumulation clustering based on the K-means algorithm. Proc of the 16th International Conference on Pattern Recognition, Quebec City.

[B16] Strehl A, Ghosh J (2002). Cluster ensembles – a knowledge reuse framework for combining multiple partitions. Journal of Machine Learning Research.

[B17] Topchy A, Jain AK, Punch W (2003). Combining multiple weak clusterings. Proc IEEE Intl Conf on Data Mining, Melbourne, FL.

[B18] Topchy A, Jain AK, Punch W (2004). A mixture model for clustering ensembles. Proc SIAM Intl Conf on Data Mining, SDM.

[B19] Fischer B, Buhmann JM (2003). Bagging for path-based clustering. IEEE Trans On Pattern Analysis and Machine Intelligence.

[B20] Dudoit S, Fridlyand J (2003). Bagging to improve the accuracy of a clustering procedure. Bioinformatics.

[B21] Chickering DM, Heckerman D (1997). Efficient approximations for the marginal likelihood of Bayesian networks with hidden variables. Machine Learning.

[B22] Peng H, Herskovits E, Davatzikos C (2002). Bayesian clustering methods for morphological analysis of MR images. Int Symp on Biomedical Imaging: from Nano to Macro, Washington, DC.

[B23] Herskovits E, Peng H, Davatzikos C (2004). A Bayesian morphometry algorithm. IEEE Transactions on Medical Imaging.

[B24] Abad PC, Lewis J, Mian IS, Knowles DW, Sturgis J, Badve S, Xie J, Lelièvre SA (2007). NuMA Influences Higher Order Chromatin Organization in Human Mammary Epithelium. Mol Biol Cell.

